# How Do Maternal Gestational Diabetes and Other Concomitant Maternal Factors Determine the Perinatal Outcomes of Pregnancy?—A Retrospective Analysis

**DOI:** 10.3390/nu17010177

**Published:** 2025-01-02

**Authors:** Karolina Karcz, Barbara Królak-Olejnik

**Affiliations:** Department of Neonatology, Wroclaw Medical University, 50-556 Wroclaw, Poland; barbara.krolak-olejnik@umw.edu.pl

**Keywords:** gestational diabetes, infant, pregnancy outcomes, neonatology, glycemic control, retrospective analysis

## Abstract

Objectives: Gestational diabetes mellitus (GDM) is associated with an increased risk of both neonatal and maternal morbidity. The aim of this retrospective study was to evaluate the frequency of perinatal complications due to GDM in the Department of Neonatology at the Medical University of Wroclaw, Poland, considering the treatment of GDM—diet and physical activity versus insulin therapy. The influence of maternal comorbidities and the COVID-19 pandemic on pregnancy outcomes was assessed. Methods: A retrospective analysis of medical records was conducted. Statistics were calculated using a range of methods, with *p* < 0.05 considered significant. A sample of *n* = 625 mothers with *n* = 646 newborns were included in this study. Results: The newborns of insulin-treated mothers had cardiovascular defects more often (*p* < 0.05). A higher prevalence of vaginal infections was found in the diet-treated mothers (*p* < 0.05), while insulin-treated mothers had a higher prevalence of pregnancy-induced hypertension, pregnancy-induced hypothyroidism and obesity (*p* < 0.05). The mode of delivery, maternal age and maternal pregnancy-induced hypertension, obesity and cholestasis were found to influence neonatal outcomes (*p* < 0.05). Conclusions: The maternal management of GDM is not the main determinant of pregnancy outcomes, which might be affected by other maternal comorbidities. Effective initiatives are needed to control GDM, support breastfeeding and prevent adverse pregnancy outcomes

## 1. Introduction

Gestational diabetes mellitus (GDM) is defined as a glucose intolerance of variable severity that begins or becomes apparent during pregnancy. This type of hyperglycemic condition presents with glucose levels between normal levels for pregnancy and glucose levels diagnostic of diabetes in non-pregnant individuals [1,2,3,4]. GDM is one of the most prevalent obstetric and metabolic complications of pregnancy, with its prevalence increasing in recent decades. GDM is estimated to affect 5–10% of pregnant women, although several studies have reported an incidence as high as 20%. The incidence of GDM varies between populations worldwide, in part due to the diagnostic approach used (e.g., 50 g or 75 g or 100 g oral glucose tolerance test) [5,6,7].

The development of insulin resistance during pregnancy is a physiological mechanism that serves to limit maternal glucose utilization, thereby ensuring an adequate supply of this energy source to the developing fetus, which relies primarily on glucose for its energy needs. Hence, maternal tissues become increasingly insensitive to insulin in a physiological pregnancy. These changes are overcome by a sufficient increase in the production of insulin by the beta cells of the pancreas [8,9]. GDM is characterized by an inadequate insulin response to compensate for the insulin resistance that results from the adaptation to the state of pregnancy. However, the precise etiology of GDM remains unclear [9,10]. Previously, it was hypothesized that insulin resistance resulted from the endogenous endocrine activity of the placenta. However, the pathophysiology of insulin resistance is complex and involves factors such as epigenetics and genetics, environmental factors, modifiable lifestyle factors and psychosocial factors. Epigenetics is typically concerned with DNA methylation patterns, whereas genetics refers to ethnicity, imprinted susceptibility or a family history of diabetes. Among environmental factors, living conditions predominate, encompassing geographical characteristics of the region and exposure to pollutants. Modifiable lifestyle factors may include dietary habits, BMI and physical activity. It is also widely acknowledged that the most influential psychosocial factors comprise depression, maternal age and maternal parity [8,10,11].

The risk of developing GDM should be assessed at the first antenatal visit. Women with clinical features suggestive of an elevated risk of GDM should undergo glucose testing as soon as possible. Therefore, immediate screening is recommended for women with severe obesity, a personal history of GDM, glycosuria or a strong family history of diabetes. At present, a 75 g glucose tolerance test is the recommended procedure for screening. If the initial screening does not indicate the presence of GDM, a subsequent test should be conducted between 24 and 28 weeks of gestation. Women at average risk should undergo screening at 24–28 weeks of gestation [1,4,5].

The increased risk of neonatal and maternal morbidity associated with GDM increases the importance of appropriate maternal management, systematic monitoring during pregnancy, optimal glycemic control and maternal and neonatal follow-up after discharge from the hospital postpartum [11]. The potential serious short-term complications of GDM include stillbirth, congenital defects, prematurity, birth trauma, hypoglycemia, dyselectrolytemia, respiratory distress, hyperbilirubinemia, polycythemia and cardiomyopathy in the newborn, as well as hypertension, pre-eclampsia, cesarean section or instrumental delivery, urinary tract infections (UTIs) or vaginal infections (VIs) and difficulty initiating or maintaining lactation in the mother. Considering long-term consequences, women diagnosed with GDM are at higher risk of developing GDM in their next pregnancy or type 2 diabetes mellitus in the next 5 to 10 years. They may also develop cardiovascular disease (hypertension, ischemic heart disease, stroke), non-alcoholic fatty liver disease, dyslipidemia or chronic kidney disease. In newborns, long-term consequences include childhood obesity or excessive abdominal adiposity, hypertension or elevated blood pressure within upper limits, metabolic syndrome, insulin resistance or early diabetes. Worldwide studies have also reported the possibility of developing autism spectrum disorders and attention deficit hyperactivity disorders [12,13,14,15].

## 2. Aim

The aim of this retrospective study was to assess the incidence of maternal and neonatal complications due to GDM in hospitalized postpartum women in the University Teaching Hospital of Wroclaw Medical University, Poland. Another objective was to compare the incidence of complications considering the GDM treatment used—diet and physical activity (GDM G1) versus insulin therapy (GDM G2). The influence of maternal comorbidities on neonatal outcomes was also one of our interests. A comparative analysis was also conducted between the results obtained prior to and following the onset of the COVID-19 pandemic.

## 3. Materials and Methods

### 3.1. Study Design

The study is a retrospective analysis of the medical records of neonates born as a result of pregnancies complicated by GDM and hospitalized after birth in the Department of Neonatology, Medical University of Wroclaw, and the medical histories of their mothers hospitalized after delivery in the 2nd Department of Gynecology and Obsterics, Medical University of Wroclaw. For the mothers, the data included age, parity, the mode of delivery, the method of maternal GDM treatment and the incidence of concomitant diseases, e.g., urinary tract infections (UTIs), vaginal infections, pregnancy-induced hypertension (PIH), chronic hypertension (HTN), hypothyroidism—chronic or in pregnancy—autoimmune disease, cholestasis of pregnancy (ICP), obesity, polycystic ovary syndrome (PCOS) or COVID-19 infection in pregnancy. The diagnosis of GDM was based on a 75 g oral glucose tolerance test (OGTT) with a 3-fold determination of plasma glucose concentration: fasting (before drinking the glucose solution) and one hour and two hours after glucose loading, according to current standards [3,4,5]. In addition to the aforementioned conditions, maternal medical records revealed the following comorbidities, which were excluded from further analysis due to their low number of cases: hepatitis C virus (HCV) infection (*n* = 1), multiple sclerosis (*n* = 1) and depressive disorder (*n* = 2). The detailed analysis of neonatal records included the incidence of congenital malformations and abnormalities found on imaging studies, assessment of anthropometric measurements (taken at birth and weight change during hospitalization), need for life support in the delivery room or provision of oxygen during the adaptation period, method of feeding the newborn, incidence of hypoglycemia, neonatal jaundice and other complications.

### 3.2. Study Group

The study group included mothers with GDM and their newborns born between 2017 and 2021. The analysis included the mothers of neonates born at term (gestational age of at least 37 + 0/7 hbd) or late preterm (gestational age between 34 + 0/7 and 36 + 6/7 hbd).

### 3.3. Data Collection

The medical histories of women with GDM and their newborns who were hospitalized in the University Teaching Hospital of Wroclaw Medical University, Poland, were analyzed.

We are an academic institution and a third-level center, the highest level of reference in south-western Poland. Our unit is responsible for a considerable number of births in the region (approximately 2000 per year), the majority of which are uncomplicated deliveries occurring within the normal gestational period. The mother and infant are typically hospitalized together in the ward for a few days, depending on the circumstances of the perinatal and early postnatal period.

**Figure 1:**
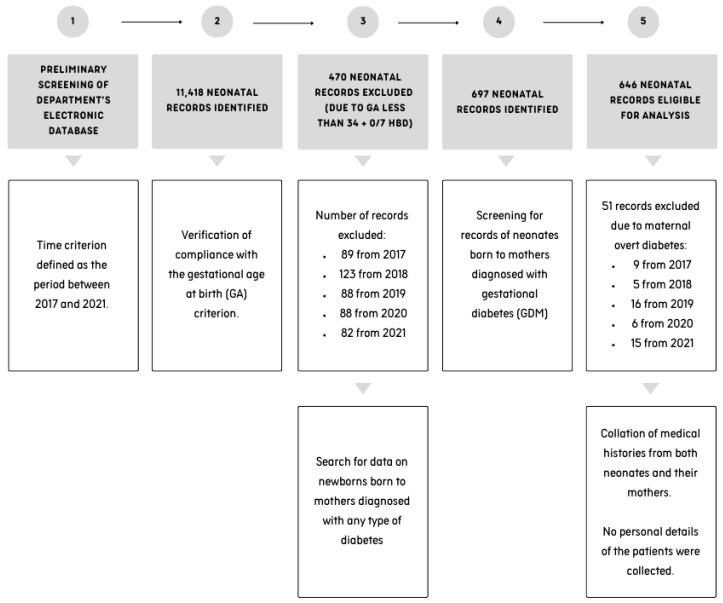
A flowchart illustrating the process of record screening.

The medical records of 11,418 newborns born to 10,964 mothers (there were 420 records of twins and 17 records of triplets) between 2017 and 2021 were screened. The search was conducted manually within the department’s electronic documentation system. The records of newborns born before the completion of 34 + 0/7 hbd (33 + 6/7 and less) were excluded from the analysis. Of the 697 records obtained, 51 were excluded due to the absence of a diagnosis of GDM during the course of pregnancy—these withheld records included data on newborns and their mothers with a diagnosis of diabetes other than GDM, such as, e.g., type 1 or type 2 diabetes. A diagrammatic representation of the data selection process is presented in Figure 1.

Anonymized data were collected in a password-protected electronic database. We had exclusive access to the database. This study did not collect any personal details that could be used to identify the patients.

### 3.4. Data Analysis

Statistica 13.3 (StatSoft, Inc., Tulsa, OK, USA) and Microsoft Excel for Office 365 (Microsoft, Redmond, WA, USA) were used for calculations. Arithmetic means, medians, standard deviations and the range of variability (extreme values) were calculated for measurable variables. For the qualitative variables, the frequency of their occurrence (percentage) was calculated. The Shapiro–Wilk test was used to determine the type of distribution of all investigated quantities. To determine differences between the study groups, a non-parametric U Mann–Whitney test was applied. The rationales for choosing this non-parametric test are as follows: (1) the need to compare data that were continuous variables with an ordinal measurement scale; (2) data distribution was not normal based on the Shapiro–Wilk test; (3) data were independent within samples (4) and a comparison of two independent groups from the same population needed to be conducted. The comparison of qualitative variables between the groups was made using a Chi-square test (χ^2^). This test was selected for analysis due to the following reasons: (1) the data subjected to analysis were categorical in nature, comprising mutually exclusive levels or categories; (2) the variables were nominal; (3) the study groups were autonomous entities and (4) there was a need to establish whether two variables were related. The assumptions required for the proper application of these tests remain consistent with the rationales for their use as described above. The application of these tests was adequate to address the posed questions and facilitate a comparative analysis of the study groups. For all calculations, *p* < 0.05 was considered significant as the significance level was set at α = 0.05.

### 3.5. Ethics

The Bioethics Committee at the Medical University in Wroclaw (No. KB 413/2020, 327/203N) expressed a favorable opinion regarding this project prior to the analysis of medical records and data collection. This opinion was rendered under the condition of anonymity with respect to the collected data. It is of the utmost importance to emphasize that at the time of admission to the Medical University Teaching Hospital, all patients were informed of the possibility of their data being potentially utilized for research purposes, provided that their personal data would be maintained in a strictly confidential and anonymized manner. The patients provided written consent in their medical records. In the case of children, consent is typically obtained from their legal representatives, most often their parents. Therefore, in the case of newborns, consent was obtained from their mothers.

## 4. Results

### 4.1. General Characteristics of Study Group

This study involved *n* = 625 GDM women who gave birth to *n* = 646 newborns (including 21 pairs of twins).

All the mothers were of the same nationality. The group exclusively comprised Polish women, who showed a lack of ethnic diversity. During the interview upon admission, the mothers expressed satisfaction with their living conditions.

The mothers provided a series of denials regarding smoking cigarettes in general, alcohol abuse prior to pregnancy and the consumption of alcohol during pregnancy. Moreover, they denied the use of other stimulants.

The mean gestational age at birth was 38.2 weeks (ranging from 34 to 42 weeks; SD = 1.5 weeks). The mean neonatal birth weight was 3304.9 g (g) (with a range of 1660.0–5230.0 g; SD = 534.0 g), while the mean neonatal length was 52.7 cm (cm) (range of 44.0–64.0 cm; SD = 3.1 cm). Most newborns were born at term (*n* = 565, 87.5%), and 52% of all newborns were boys. The overwhelming majority of newborns, representing more than 90% of cases, were born in a good condition. The Apgar scores at the 1st, 5th and 10th minutes were as follows: the median was 9, range of 1–10 points; the median was 10, range of 4–10; and the median was 10, range of 6–10, respectively.

The vast majority of women had a cesarean section (70.6%). Newborns delivered by cesarean section were hospitalized with their mothers for a longer period of time than those born naturally (*p* < 0.05), 4.7 (SD = 4.1) vs. 4.1 (SD = 2.9), respectively. The former group of both mothers and newborns more often required additional care and observation. A total of 6.5% of the newborns were from twin pregnancies (*n* = 42 newborns).

Of the mothers with GDM, 61.4% were treated with insulin (GDM G2 group), while the remaining 38.6% were treated with diet and lifestyle changes (GDM G1 group). The mean daily insulin dose was 17.8 units (min–max: 2.0–120.0 units; SD = 15.5 units)

The mean age of the mothers on the day of delivery was 33 years (min–max: 19–45 years; SD = 4.5 years), with a mean age of 32.5 years (min–max: 20–45 years, SD = 4.5) in the GDM G1 group and a mean age of 33.4 years (min–max: 19–44 years, SD = 4.3) in the GDM G2 group—these results were found to be significant (*p* < 0.05). The mean number of pregnancies was 2.1 (min–max: 1.0–9.0; SD = 1.2), and the mean number of deliveries was 1.7 (min–max: 1.0–6.0; SD = 0.8), with nonsignificant difference between the study groups (*p* > 0.05).

In both the G1 and G2 groups, the highest number of newborns were born in the years 2019–2020. The detailed results are shown in Table 1.

### 4.2. Maternal GDM and Other Maternal Comorbidities

**Figure 2:**
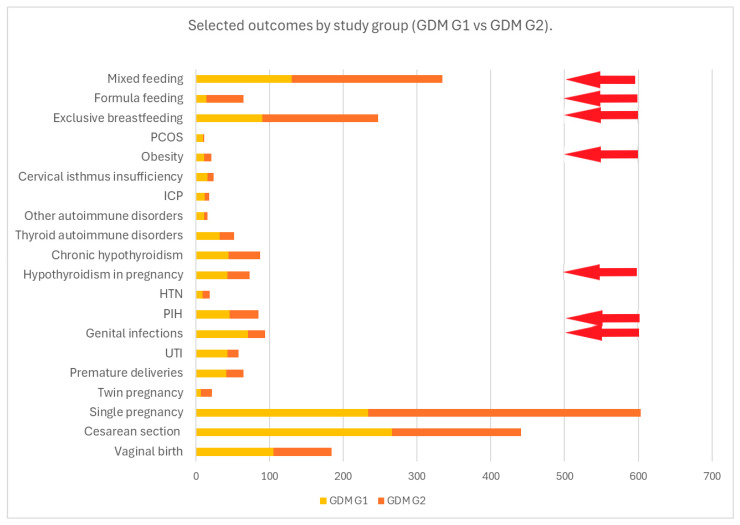
This figure presents the frequency of the selected outcomes compared by study group (GDM G1 vs. GDM G2). The red arrows indicate statistically significant results for *p* < 0.05.

Maternal medical history was analyzed and compared according to the type of GDM treatment. Mothers treated with insulin had a significantly higher prevalence of PIH, hypothyroidism in pregnancy and obesity (*p* < 0.05), whereas a significantly higher prevalence of vaginal infections was found in mothers treated with diet and physical activity—Table 2 and Figure 2.

### 4.3. Maternal Comorbidities During Pregnancy and Neonatal Outcomes

#### 4.3.1. Respiratory Support

Regarding the effects of maternal comorbidities on neonatal outcome, neonates whose mothers suffered from PIH, ICP and obesity were significantly more likely to require support of transition at birth (*p* < 0.05). Table 3 shows a comparison of the results for maternal complaints due to the need for respiratory support for the newborn in the delivery room. The age of the mother and the method of delivery also had a significant impact on this neonatal complication. Women whose newborns were in need of ventilation were significantly younger than the mothers of babies with uncomplicated transition (mean age of 31.8 years SD = 4.4 years, min–max: 19–41 years versus mean age of 33.3 years SD = 4.4 years, min–max: 20–45 years, *p* < 0.001). Furthermore, infants delivered via caesarean section demonstrated a higher likelihood of requiring ventilation (breathing assistance was required in 15.6% of newborns delivered by cesarean section and 9% of those delivered via vaginal birth, *p* < 0.001).

#### 4.3.2. Hypoglycemia

**Figure 3:**
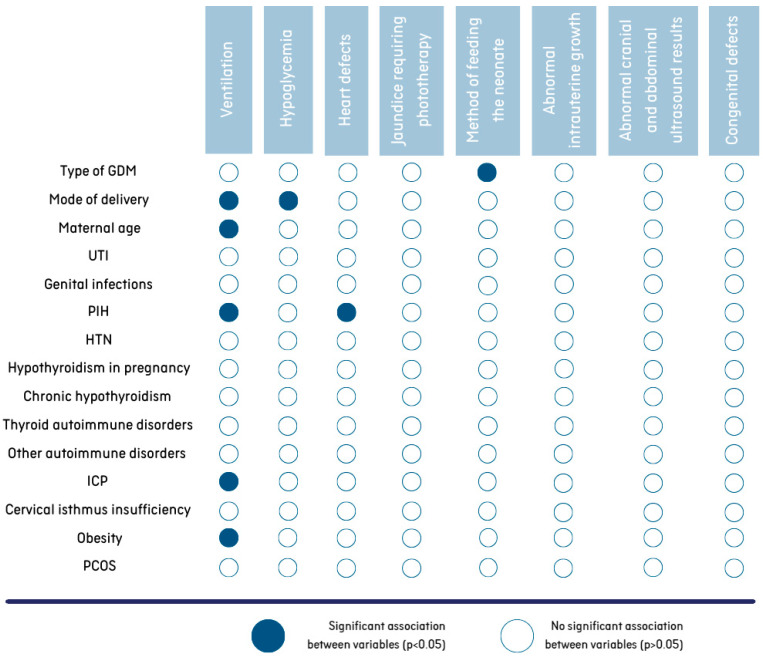
Associations between maternal characteristics/morbidities and neonatal outcomes.

It was found that hypoglycemia was more common in newborns born by cesarean section (*n* = 82, 17.8%) than in those born by vaginal birth (*n* = 18, 9.8%) (*p* = 0.01) (Figure 3).

#### 4.3.3. Cardiovascular Outcomes

An analysis of the data shows that mothers with PIH are more likely to give birth to children with heart defects than mothers with normal blood pressure during pregnancy (*p* < 0.05). A total of 28.1% of newborns diagnosed with a heart defect were born to mothers with PIH (Figure 3). Among neonates without cardiovascular problems, 12.7% were born from PIH-complicated pregnancies.

#### 4.3.4. Other Outcomes

No differences (*p* > 0.05) were found in the prevalence of other complications in the newborns (e.g., jaundice requiring phototherapy, abnormal intrauterine growth, abnormal cranial and abdominal ultrasound results, congenital defects) depending on the presence of other complaints in the mothers (Figure 3).

### 4.4. Effects of COVID-19 Pandemic on Maternal Outcomes

Of all the mothers, only *n* = 8 had SARS-CoV-2 infection during pregnancy—all cases were in 2021. Seven of these were in the GDM G2 group, and one was in the GDM G1 group. Further analysis was not performed because of the small number of cases.

Before and during the pandemic, GDM patients were just as likely to be diet-controlled and insulin-treated (*p* > 0.05). There was no significant difference (U Mann–Whitney test, *p* > 0.05) in the daily dose of insulin in the GDM G2 group before (mean insulin dose 17.7 units, SD = 18.4 units, min–max: 2.0–120.0 units) or during the pandemic (mean insulin dose 18.2 units, SD = 12.3 units, min–max: 2.0–58.0 units).

In a detailed analysis of the prevalence of comorbidities, PCOS and genitourinary tract infections were less common in mothers during the pandemic (*p* < 0.05). No difference was observed in the prevalence of other concomitant diseases (e.g., PIH or HTN, autoimmune disorders, hypothyroidism, ICP, cervical isthmus insufficiency, obesity) (*p* > 0.05).

During the pandemic, mothers with GDM were just as likely as before the pandemic to have a cesarean section, to have a preterm birth or to give birth to a baby with abnormal intrauterine growth (SGA or LGA) (*p* > 0.05)

However, there was an increased prevalence of the formula feeding of newborns and a lower rate of exclusive breastfeeding among mothers with GDM during the pandemic (*p* < 0.05).

**Figure 4:**
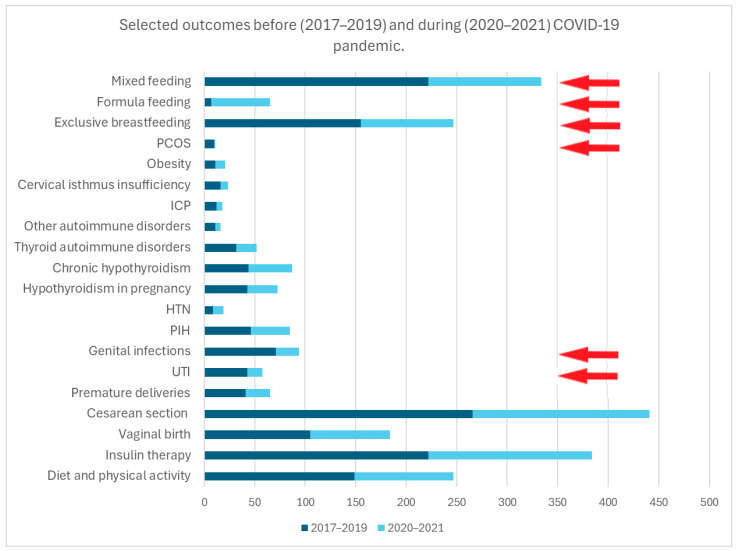
This figure presents the frequency of selected outcomes, comparing the period prior to the onset of the pandemic (years 2017–2019) with the period during the pandemic (years 2020–2021). The red arrows indicate statistically significant results for *p* < 0.05.

The results are presented in Table 4 and Figure 4.

In 2020, approximately 35.2% of mothers reported experiencing difficulties in scheduling appointments with healthcare providers in a conventional face-to-face setting. In the course of the interview, the mothers conceded that the most prevalent source of medical information to which they had recourse was the internet. A positive change was observed in 2021, the second year of the pandemic, which followed the suspension of regional prohibitions. Nonetheless, there is an absence of data concerning the availability of perinatal care in the years 2017–2019 prior to the pandemic. It is important to note that a lactational consultation was made available to all mothers during postpartum hospitalization, as a standard procedure in our department.

## 5. Discussion

A review of data from 2017 to 2021 revealed a notable presence of GDM among mothers who were hospitalized in the University Teaching Hospital of Wroclaw Medical University, Poland. In nearly every year, the count of GDM patients was greater than that of the previous year, with the majority of women receiving insulin therapy. In consideration of the percentage of cases of GDM, the reporting rate is 4–9% per year, which is consistent with the current data from Europe and the rest of the world [5,6,7,16]. In light of this observation, it can be inferred that the outcomes of our study are comparable to those of the general population.

The prevalence of GDM in Europe was found to be increased in pregnant women aged > 30 years [16]. Maternal age is therefore associated with the overall risk of glucose metabolism disorders [10,11,12]. The present study included mothers with GDM treated with diet (G1) and insulin (G2)—the average maternal age in both groups was over 30 years and was significantly higher in the G2 group. This observation is an indication that the association between maternal age and impaired glucose metabolism is true and replicable.

It is well known that obesity is associated with insulin resistance, which puts women at risk of developing GDM during pregnancy [15]. Obese women were more likely to have a higher degree of impaired glucose metabolism—this finding is consistent with studies worldwide [11,12]. In addition, gravidas with a higher degree of carbohydrate metabolism disorders requiring insulin therapy had a higher prevalence of developing genitourinary tract infections, hypertension or hypothyroidism with onset during pregnancy. These complications have been reported frequently worldwide over the past decade [17,18,19]. Therefore, the incidence of respiratory distress was higher among the newborns of the abovementioned mothers, which is consistent with the findings of previous studies [13,14,15,19].

The analysis revealed that a considerable proportion of the women in the study group underwent caesarean sections. Indeed, our unit has a high prevalence of cesarean sections, which are performed due to maternal or fetal emergencies as well as ineffective labor induction. Furthermore, studies from around the world have indicated a high prevalence of cesarean sections due to maternal or fetal condition [19,20].

The results on breastfeeding rates deserve special attention. A significantly elevated prevalence of formula feeding was observed in the postpartum period, particularly within the GDM G2 cohort, accompanied by a notable reduction in exclusive breastfeeding during the pandemic. In general, women with GDM have been observed to experience difficulties in breastfeeding their infants, with lower rates of successful breastfeeding initiation compared to women without GDM. Based on evidence, they are more likely to have a delayed onset of lactogenesis II. As women with GDM report more difficulty producing enough milk than women without diabetes, the infants of mothers with GDM are introduced to fluids other than human milk (e.g., formula) earlier than the infants of women without diabetes. The introduction of formula is associated with the earlier cessation of breastfeeding in some women [21,22,23,24]. Taking all these issues into account, mothers with GDM should receive specific breastfeeding support, which may improve their breastfeeding performance.

The production of insulin by the fetus is contingent upon the provision of glucose through the placenta. A direct relationship exists between maternal hyperglycemia and fetal hyperglycemia, which in turn results in fetal hyperinsulinemia. Following delivery, when the glucose supply is ceased, the newborn is at risk of developing hypoglycemia due to the elevated insulin concentration that persists in the absence of glucose [11,12,13]. In this study, the incidence of hypoglycemia was higher in neonates born by cesarean section, which may be attributed to delayed first feeding and greater difficulty in stimulating lactation in these mothers.

The COVID-19 pandemic outbreak in 2020 resulted in sudden restrictions on outpatient healthcare in Poland. These included pregnancy management. Based on regulations implemented by the Government and the National Health Fund, outpatient clinics offered teleconsultations instead of in-person appointments to ensure the continuity of care for their patients [25]. There are questions about the quality and adequacy of the medical advice given to patients. Except for breastfeeding rates, the results of the present study did not differ when compared before and after the COVID-19 pandemic outbreak. On the one hand, this may be related to well-organized remote care, and on the other hand, some mothers with complications during pregnancy may have been under-diagnosed.

A discussion is required regarding the potential factors contributing to the observed decline in breastfeeding rates. The primary hypothesis we propose is that the challenges encountered in the implementation of office-based appointments, which had consequences for the entire healthcare sector due to the regulations of the lockdown, may have precipitated a decline in maternal preparedness. This decline can be attributed to the suboptimal antenatal education that resulted from the reduced number of midwifery consultations. The subsequent hypothesis was formulated on the basis of empirical findings derived from our clinical work. The mothers expressed a strong desire to be discharged from the hospital as expeditiously as possible. The mothers were concerned that their newborn baby might experience excessive weight loss or jaundice, necessitating phototherapy, which would result in an extended stay in the facility. For this reason, they were eager to administer a milk formula to the infant. Simultaneously, they asserted that it would be more expedient for them to stimulate lactation and undertake breastfeeding in their domestic environment.

Current evidence shows conflicting results about the relationship between GDM and particular adverse pregnancy outcomes. However, GDM is a broad category of maternal hyperglycemia in which blood glucose levels correlate with a wide range of metabolic abnormalities and confer varying degrees of risk for pregnancy-related complications [8,9,10,26]. Worldwide studies have confirmed the link between GDM and adverse maternal outcomes, including PIH (thus pre-eclampsia) and cesarean section. Pre-eclampsia, characterized by high blood pressure due to increased insulin resistance, is a common pregnancy complication caused by GDM. The incidence of hypertensive disorders can increase by two to three times during pregnancy due to high blood glucose levels. However, several other maternal factors are also associated with pre-eclampsia, including maternal cardiovascular disease, renal disease and overweight and obesity [27,28]. Maternal hypertension and pre-eclampsia are risk factors for neonatal respiratory disease in both term and preterm infants [29]. In addition, based on available studies, it has been found that the babies of obese mothers are more likely to develop respiratory distress than the babies of non-obese mothers [30,31]. When these relationships are considered together, it can be concluded that the maternal factor has an additive effect on the outcome of pregnancy.

The findings of this study offer invaluable insights for healthcare professionals seeking to implement efficacious strategies to enhance GDM-related care for women of reproductive age, both before and after conception. This is not merely a matter of addressing GDM but also of preventing adverse pregnancy outcomes. It can thus be concluded that the control of GDM may act as an indirect preventive measure against adverse pregnancy outcomes and future long-term maternal and neonatal morbidity.

## 6. Strengths

First of all, this present retrospective analysis compared outcomes among mothers diagnosed with GDM between the group treated with diet and physical activity (GDM G1) and the group treated with insulin (GDM G2). To date, most studies have not distinguished between mothers based on the method of treatment to achieve glycemic control. It is our considered opinion that this analysis was necessary to compare the results not strictly in terms of treatment but also in regard to the degree of impaired glucose metabolism.

In addition, the perinatal outcomes of newborns were compared due to other complications/conditions present in the mother—allowing us to identify a group of newborns with a higher risk of complications of the postpartum period.

Furthermore, the results for mother–child pairs obtained before the outbreak of COVID-19 and during the first 2 years of the pandemic were compared. At that time, care for pregnant women, including those with GDM, deteriorated due to national restrictions.

To the best of our knowledge, this is one of the first studies to assess the impact of the first years of the COVID-19 pandemic on the perinatal outcomes of mothers diagnosed with GDM and their newborn infants.

## 7. Limitations

The following limitations apply to the present study. Analyzing patients’ records retrospectively can suffer from bias due to recall bias or misclassification. A dearth of data pertaining to pregnancy exists within the hospital’s electronic records. This includes the precise outcomes of diagnostic tests, weight gain and information regarding continuous obstetric surveillance and its adherence to standards of practice. A comprehensive maternal history typically encompasses the following elements: a detailed account of the patient’s pregnancy, a comprehensive account of chronic and pre-pregnancy illnesses, obstetric history, the results of tests conducted during hospitalization and the details of the hospitalization course. The initial medical entry comprises solely annotations pertaining to the completion of requisite tests and noteworthy abnormalities observed in their outcomes. In consequence of the aforementioned circumstances, a retrospective analysis of the pregnancy card and a detailed review of the diagnostic tests conducted during the gestational period were not feasible.

Maternal records did not include OGTT results, so the maternal diagnosis of GDM was assumed to be adequate based on the annotations made during the medical interview. In addition, retrospective analyses provide a lower level of evidence than prospective studies. The effectiveness of maternal glycemic control could not be assessed—we did not have information on maternal glycemia or at least the frequency of hyperglycemia. Other risk factors that were not measured may have been present in the study group—for example, we were not able to assess the influence of maternal BMI, gestational weight gain, percentage of glycated hemoglobin or the results of laboratory tests such as a lipid profile on the outcomes recorded in both mothers and newborns.

## 8. Conclusions

In the case of effectively controlled gestational diabetes, the chosen treatment method does not have an impact on the perinatal outcomes of newborns. Gravidas with a higher degree of glucose metabolism disorders requiring insulin therapy have a higher risk of developing other complications during pregnancy. It is crucial to acknowledge that other pre-pregnancy complications (such as obesity) and pregnancy-related issues (gestational cholestasis, pregnancy-induced hypertension) also influence neonatal outcomes. However, women treated with insulin are at an elevated risk of experiencing lactation failure. Despite the decline in outpatient care accessibility during the initial stages of the pandemic, the incidence of caesarean section deliveries, preterm births and abnormal fetal growth among women diagnosed with gestational diabetes remained comparable to previous years. There is a need to set targets for interventions to improve breastfeeding and, ultimately, long-term maternal and newborn health. Further research is needed to assess the cumulative effects of maternal comorbidities on the course of gestational diabetes and pregnancy outcomes. Further investigations should focus on genetic and environmental factors and their ability to modify maternal and neonatal outcomes in pregnancies complicated by GDM. Effective initiatives should be taken to control GDM and thus prevent adverse pregnancy outcomes and morbidity in the population affected by GDM.

## Figures and Tables

**Table 1:** Maternal characteristics by type of GDM.

Study Group (*n* = 625)											
	GDM G1					GDM G2					*p* Value
	$\bar{x}$	Me	Min	Max	SD	$\bar{x}$	Me	Min	Max	SD	
Age [years]	32.5	33.0	20.0	45.0	4.5	33.4	33.5	19.0	44.0	4.3	0.021 *
Gravidity	2.1	2.0	1.0	7.0	1.1	2.2	2.0	1.0	9.0	1.2	0.193 *
Parity	1.7	2.0	1.0	4.0	0.8	1.8	2.0	1.0	6.0	0.8	0.051 *
Duration of pregnancy [weeks of gestation/hbd]	38.3	38.0	34.0	42.0	1.5	38.2	38.0	34.0	42.0	1.4	0.609 *
	*n* (%)					*n* (%)					
Years											
2017	38 (16)					47 (12)					<0.001 **
2018	33 (14)					85 (22)					
2019	78 (32)					90 (23)					
2020	73 (30)					86 (22)					
2021	19 (8)					76 (20)					
Mode of delivery											
Vaginal birth	77 (32)					107 (28)					0.276 **
Cesarean section	164 (68)					277 (72)					
Number of fetuses											
Single	234 (97)					369 (96)					0.508 **
Twin	7 (3)					15 (4)					

*n*—number; %—percent; $\bar{x}$—mean; Me—median; Min—minimal value; Max—maximal value; SD—standard deviation; * *p* value based on U Mann–Whitney test; ** *p* value based on χ^2^ test. The bold indicates statistically significant *p*-values.

**Table 2:** Comparison of results on prevalence of maternal comorbidities due to treatment of GDM.

Maternal Concomitant Diseases		GDM G1		GDM G2		*p* Value *
		*n*	%	*n*	%	
UTI	Yes	26	9.4	36	9.7	0.915
Genital infections	Yes	51	18.6	46	12.4	**0.031**
PIH	Yes	24	8.7	63	17.0	**0.002**
HTN	Yes	11	4.0	9	2.4	0.253
Hypothyroidism in pregnancy	Yes	22	8.0	55	14.8	**0.008**
Chronic hypothyroidism	Yes	33	12.0	60	16.2	0.135
Thyroid autoimmune disorders	Yes	25	9.1	28	7.6	0.480
Other autoimmune disorders	Yes	10	3.6	6	1.6	0.103
ICP	Yes	11	4.0	9	2.4	0.253
Cervical isthmus insufficiency	Yes	13	4.7	14	3.8	0.549
Obesity	Yes	5	1.8	18	4.9	**0.040**
PCOS	Yes	6.0	1.2	6.0	2.2	0.599

*n*—number; %—percent; * χ^2^ test; UTI—urinary tract infection; PIH—pregnancy-induced hypertension; HTN—chronic hypertension; ICP—cholestasis of pregnancy; PCOS—polycystic ovary syndrome. The bold indicates statistically significant *p*-values.

**Table 3:** Comparison of results on need for respiratory support in newborns at birth due to maternal complaints.

Maternal Concomitant Diseases		The Respiratory Support of the Newborn in the Delivery Room				*p* Value *
		No		Yes		
		*n*	%	*n*	%	
UTIs	Yes	53	9.4	9	10.8	0.680
Genital infections	Yes	87	15.5	10	12.0	0.418
PIH	Yes	70	15.6	17	20.5	**0.045**
HTN	Yes	16	2.8	4	4.8	0.332
Hypothyroidism in pregnancy	Yes	67	11.9	10	12.0	0.969
Chronic hypothyroidism	Yes	77	13.7	16	19.3	0.175
Thyroid autoimmune disorders	Yes	45	8.0	8	9.6	0.610
Other autoimmune disorders	Yes	13	2.3	3	3.6	0.475
ICP	Yes	13	2.3	7	8.4	**0.003**
Cervical isthmus insufficiency	Yes	21	3.7	6	7.2	0.137
Obesity	Yes	17	3.0	6	7.2	**0.049**
PCOS	Yes	11	2.0	1	1.2	0.637

*n*—number; %—percent; * χ^2^ test; UTIs—urinary tract infections; PIH—pregnancy-induced hypertension; HTN—chronic hypertension; ICP—cholestasis of pregnancy; PCOS—polycystic ovary syndrome. The bold indicates statistically significant *p*-values.

**Table 4:** Comparison of selected outcomes before (2017–2019) and during (2020–2021) COVID-19 pandemic.

Study Group (*n* = 625 Mothers/*n* = 646 Neonates)			
	Years		*p* Value *
	2017–2019	2020–2021	
	*n* (%)	*n* (%)	
Treatment of GDM			
Diet and physical activity	149 (40.0)	98 (38.0)	0.320
Insulin therapy	222 (60.0)	162 (62.0)	
Mode of delivery			
Vaginal birth	105 (28.0)	79 (31.0)	0.451
Cesarean section	266 (72.0)	175 (69.0)	
Neonatal birth weight to gestational age			
SGA	19 (4.9)	10 (3.8)	0.448
AGA	309 (80.5)	221 (84.4)	
LGA	56 (14.6)	31 (11.8)	
Method of feeding neonate			
Exclusive breastfeeding	155 (40.4)	92 (35.1)	**<0.001**
Formula feeding	7 (1.8)	58 (22.1)	
Mixed feeding	222 (57.8)	112 (42.8)	
Premature deliveries	41 (11.0)	24 (9.0)	0.519
UTIs	43 (12.0)	15 (6.0)	**0.016**
Genital infections	71 (19.0)	23 (9.0)	**<0.001**
PIH	46 (12.0)	39 (15.0)	0.290
HTN	9 (2.0)	10.0 (4.0)	0.280
Hypothyroidism in pregnancy	43 (12.0)	30 (12.0)	0.933
Chronic hypothyroidism	44 (12.0)	43 (17.0)	0.072
Thyroid autoimmune disorders	32 (9.0)	20 (8.0)	0.738
Other autoimmune disorders	11 (3.0)	5 (2.0)	0.438
ICP	12 (3.0)	6 (2.0)	0.522
Cervical isthmus insufficiency	16 (4.0)	8 (3.0)	0.457
Obesity	11 (3.0)	10 (4.0)	0.510
PCOS	10 (3.0)	1 (0.0)	**0.032**

*n*—number; %—percent; * *p* value based on χ^2^ test; UTIs—urinary tract infections; PIH—pregnancy-induced hypertension; HTN—chronic hypertension; ICP—cholestasis of pregnancy; PCOS—polycystic ovary syndrome; AGA—appropriate for gestational age; LGA—large for gestational age; SGA—small for gestational age. The bold indicates statistically significant *p*-values.

## Data Availability

The original contributions presented in this study are included in the article; further inquiries can be directed to the corresponding author.
